# The Psychometric Properties of the French–Canadian Stress and Anxiety to Viral Epidemics-6 Scale for Measuring the Viral Anxiety of the General Population During the COVID-19 Pandemic

**DOI:** 10.3389/fpsyt.2022.807312

**Published:** 2022-03-31

**Authors:** C. Hyung Keun Park, Oli Ahmed, Sangha Lee, Sooyeon Suh, Seockhoon Chung, Jean-Philippe Gouin

**Affiliations:** ^1^Department of Psychiatry, Asan Medical Center, University of Ulsan College of Medicine, Seoul, South Korea; ^2^Department of Psychology, University of Chittagong, Chattogram, Bangladesh; ^3^National Centre for Epidemiology and Population Health, Australian National University, Canberra, ACT, Australia; ^4^Department of Psychiatry, Ajou University School of Medicine, Suwon, South Korea; ^5^Department of Psychology, Sungshin Women's University, Seoul, South Korea; ^6^Department of Psychology, Concordia University, Montreal, QC, Canada

**Keywords:** health personnel, COVID-19, SAVE-6, anxiety, stress

## Abstract

**Objective:**

This study examined the psychometric properties of the French–Canadian version of the Stress and Anxiety to Viral Epidemics-6 items (SAVE-6) scale for assessing the anxiety response to the viral epidemic among the general population in Quebec, Canada.

**Methods:**

A total of 590 participants responded to a confidential online survey between September 28 and October 18, 2020. Confirmatory Factor Analysis (CFA) was conducted to explore the factor structure of the scale. Psychometric properties were assessed using the Item Response Theory (IRT) approach. To explore the convergent validity, a Pearson correlation analysis between the SAVE-6 scale and the depression (Patient Health Questionnaire-2, PHQ-2) or anxiety subscale (Generalized Anxiety Disorder-2, GAD-2) of the Patient Health Questionnaire-4 items scale was conducted.

**Findings:**

The French–Canadian version of the SAVE-6 scale was clustered into a single factor. The CFA of the SAVE-6 scale showed a good model fit (CFI = 0.985, TLI = 0.976, RMSEA = 0.051, RSM*R* = 0.048), and the multi-group CFA revealed that the SAVE-6 scale can measure anxiety response in the same way across gender or the presence of elevated depressive and anxiety symptoms. It showed good internal consistency (Cronbach's alpha = 0.76, McDonald's Omega = 0.77) and significant correlation with the PHQ-2 score and GAD-2 score. The IRT model suggested the efficiency in discrimination among individuals in this latent trait.

**Conclusion:**

The French–Canadian version of the SAVE-6 scale is a valid and reliable rating scale, which can measure the general population's anxiety response to the viral epidemic.

## Introduction

Since the beginning of the twenty-first century, the world has suffered from outbreaks of various infectious diseases—the severe acute respiratory syndrome outbreak in 2002, the H1N1 influenza pandemic in 2009, the Middle East respiratory syndrome outbreak in 2012, and the ongoing coronavirus disease 2019 (COVID-19) pandemic. These outbreaks have seriously impacted not only physical health but also on mental health; psychological responses such as anxiety, depression, and posttraumatic stress were reported in the general population (1). Various aspects of an infectious disease outbreak, such as disruptions in routine and lifestyle (2), stigmatization (3), quarantine (4), and fear of abandonment (2), may contribute to the impairment of psychological well-being.

In Canada, the first COVID-19 infection was transmitted by an individual who had visited Wuhan, Hubei, China, and tested positive for SARS-CoV-2 on January 27, 2020 (5). The first community transmission was detected in March 2020, and, since then, there have been confirmed cases in each Canadian province and territory (5). As of October 4, 2021, COVID-19 has affected 1,640,606 people nationwide; more than 28,000 people have died from the disease, and 1,568,285 have recovered (6). Despite the fact that the province of Quebec accounts for only one-fourth of the total Canadian population, over 40% of COVID-19-related deaths have occurred there (6). This may be due to high infection rate among seniors residing in long-term care facilities as well as cultural difference in compliance with COVID-19 restrictions (5).

Since the COVID-19 pandemic is an infectious disease outbreak, the general population has experienced mental health problems similar to those seen in previous pandemics. Many studies worldwide have shown that, during the pandemic, the point prevalence of psychiatric symptoms—moderate to severe depression and anxiety disorder—has been higher than usual (7). Likewise, Canadians also have shown symptoms of mental health deterioration (7). Specifically, the proportion of those detected with generalized anxiety disorder and depression has increased, and an increase in alcohol and cannabis use has been reported (7). Another survey indicated the worsening of mental health in over 50% of respondents sampled from the Canadian general population during the pandemic (8).

As a more serious matter, the COVID-19 outbreak may have increased suicide risk through various factors related to stress and anxiety; these include (1) the uncertain nature of the potentially lethal disease, (2) social isolation from lockdown and social distancing, and (3) economic hardship caused by the loss of jobs (9). The decline in psychiatric care is also associated with increased suicidal ideation (7). In addition, these psychological responses can endure even after the pandemic (9).

Given the psychological impact of the pandemics, it is meaningful to develop a rating scale by which we can measure the anxiety responses specific to viral pandemics. During the COVID-19 pandemic, several rating scales were developed for this purpose such as the 36-item COVID Stress Scales (10), the Fear of COVID-19 Scale (11), the Coronavirus Anxiety Scale (12), the COVID-19-Anxiety Questionnaire (13), the Obsession with COVID-19 Scale (14), the Coronavirus Pandemic Anxiety Scale (15), the COVID-19 Anxiety Syndrome Scale (16), and the COVID-19 Anxiety Scale (17). We developed the Stress and Anxiety to Viral Epidemics-6 item (SAVE-6) to measure the anxiety responses of the general population and validated it among Korean (18), Arabic (19), and American (20) samples. Additionally, the SAVE-6 scale was examined for special populations such as medical students (21), public workers (22), and cancer patients (23). In this study, we explored the factorial validity of the French–Canadian version of the SAVE-6 scale and evaluated whether it can measure the anxiety responses of the French-speaking Canadian sample.

## Materials and Methods

### Participants and Procedure

This study is a part of a longitudinal study of 1,003 participants, representative of the adult (18 years and older) population of Quebec, Canada (24). It was conducted in three waves—the first wave took place between April 7 and 15, 2020 (Time 1), the second wave between May 19 and June 7, 2020 (Time 2), and the third wave between September 28 and October 18, 2020 (Time 3). An invitation email to complete the survey was sent to all 21,885 individuals within a web panel who were at least 18 years old and currently living in Quebec. Of the 1,701 individuals who read the consent, 59% agreed to participate in this study and fully completed the survey. This validation study used the time 3 data collected at the start of the second wave of the pandemic when retail and restaurant closures and part-time virtual schooling for high school students were reintroduced (25). Among the 1,003 baseline (Time 1) participants, 650 completed the Time 3 survey Excluding 60 participants who responded to the English version of this survey, all 590 participants who responded to the French–Canadian version of this survey were used in this validation study. This study was approved by the Concordia University institutional ethics review board (#30012927).

### Assessment

#### Sociodemographic and Medical Characteristics

Participants' information regarding age, sex, level of education, household composition, and essential worker status were gathered. Participants also responded to the questions regarding whether they had a physical health problem that can increase their susceptibility to severe COVID-19.

#### Stress and Anxiety to Viral Epidemics-6 Items Scale

The SAVE-6 scale is a self-rating scale for measuring respondents' anxiety responses specifically to the viral epidemic (18). The six items can each be rated on a Likert scale ranging from 0 (never) to 4 (always). The total score ranges from 0 to 24, with higher scores reflecting higher levels of anxiety in response to the viral epidemic. This scale was drawn from the SAVE-9 scale (26), which was originally developed to measure the work-related stress and anxiety responses of healthcare workers to the viral COVID-19 pandemic. The SAVE-9 scale was clustered into two factors; factor I - SAVE-6 (item 1, 2, 3, 4, 5, and 8) (18) and factor II—SAVE-3 (item 6, 7, and 9)(27). We developed the SAVE-6 as a tool for measuring the viral anxiety of the general population, and SAVE-3 as a brief scale to measure work-related stress of healthcare workers. The questionnaire was translated using a back-translation method. Two bilingual experts translated the questionnaire into the French–Canadian version from the English version. Then these two translated French–Canadian versions were synthesized into one. Next, this synthesized version was back translated into English by two other bilingual experts. These two back translations were again synthesized into one and compared with the original English version to identify any discrepancy in meaning. As there was no discrepancy in meaning, the translated French–Canadian version was used for data collection.

#### Patient Health Questionnaire-4 Items Scale

The Patient Health Questionnaire-4 items (PHQ-4) scale is an ultra-brief rating scale for measuring patients' depression and anxiety (28). It was derived from the Patients Health Questionnaire-9 (PHQ-9) (29) and Generalized Anxiety Disorder-7 (GAD-7) (30) scales, which are designed to measure the severity of depression (29) and generalized anxiety (30). It consists of four items, which includes two items from the Patient Health Questionnaire-2 items (PHQ-2) screening tool (31) for depression and two items from the Generalized Anxiety Disorder-2 items (GAD-2) screener (32). Each item can be rated on a four-point Likert scale (0 for not at all, 1 for several days but <1 week, 2 for more than half the time, and 3 for nearly every day). Depression and generalized anxiety were indicated by scores ≥3 for PHQ-2 and GAD-2, respectively. The Cronbach's alpha of PHQ-4 scale was 0.877 for this sample. Specifically, the split-half values of PHQ-2 and GAD-2 were 0.879 and 0.846.

### Statistical Analysis

First, we conducted the initial screening test and exploratory factor analysis with oblimin rotation to explore the factor dimension of the SAVE-6 scale and parallel analysis (33), based on Maximum Likelihood, with a 95 percentile threshold based on the reduced correlations matrix; this was conducted to determine the number of factors to be retained for the adapted SAVE-9 and SAVE-6 scales, using the *nFactor* R package. Model fits were assessed through root mean square of the residuals [SRMR], and root-mean-square-error of approximation [RMSEA]. The factor model was constructed by comparing the real-data eigenvalues that exceeded the 95th percentile of the random eigenvalues. Prior to the statistical analysis, the normality consumption was checked based on the skewness and kurtosis of values within the range ± 2 (34). Data suitability and sampling adequacy were checked using the Kaiser-Meyer-Olkin (KMO) value and Bartlett's test of sphericity. Second, a confirmatory factor analysis (CFA) [estimation method = diagonal weighted least square] was conducted to explore the construct validity of the French–Canadian version of the SAVE-6 scale. Satisfactory model fit was defined by a standardized root-mean-square residual (SRMR) value ≤ 0.05, a root-mean-square-error of approximation (RMSEA) value ≤ 0.10, and comparative fit index (CFI) and Tucker Lewis index (TLI) values ≥ 0.90 (35, 36). A multi-group CFA with configural invariance testing was conducted to examine whether the SAVE-6 scale could measure anxiety responses in the same way among respondents with depression (PHQ-2 ≥ 3) or anxiety (GAD-2 ≥ 3), across any gender, across respondents who knew or did not know someone diagnosed with COVID-19, and across respondents who do or do not work with a greater risk of exposure. Psychometric properties were also assessed using the Item Response Theory (IRT) approach (graded response model [GRM]). Before running the GRM, IRT assumptions—unidimensionality, local dependence, and monotonicity—were assessed. The GRM provided slope/discriminating parameters and threshold/difficulty parameters for items. Item fit statistics were also estimated. Unidimensionality and monotonicity were assessed using the R package mokken version 3.0.6, and local dependance and slope and threshold parameters in the GRM were estimated using the R package mirt version 1.34. Third, the reliability and internal consistency of the scales were assessed using Cronbach's alpha and McDonald's Omega. To explore the convergent validity, a Pearson correlation analysis was conducted between the SAVE-6 scale and the depression or anxiety subscale from the PHQ-4 scale. The SPSS version 21.0, AMOS version 27 (SPSS, Inc, Chicago, Illinois), JASP version 0.14.1.0 software (JASP Team, Amsterdam, Netherlands), and RStudio were used for statistical analysis.

## Results

### Demographic Characteristics

In total, 590 participants responded to the French–Canadian version of the survey. Among them, 51.9% were female (Table 1), 20.3% were living alone, 39.2% had one or more COVID-19-specific health risk(s), 13.1% knew someone diagnosed with COVID-19, 16.3% were essential workers at greater risk of SARS-CoV-2 exposure, and 6.1% were healthcare workers. Participants' mean age was 52.4 ± 15.1 years old, 11.7% were assessed as having depression (PHQ-2 ≥ 3), and 18.1% were assessed as having anxiety (GAD-2 ≥ 3).

**Table 1:** Demographic characteristics of the participants (*N* = 590).

**Variables**	***N* (%) or Mean ±SD**
**Female**	306 (51.9%)
**Regions**	
Grande région de Montréal	279 (47.3%)
Grande région de la Ville de Québec	69 (11.7%)
Ailleurs au Québec	242 (41.0%)
**Age (years)**	52.4 ± 15.1
18–29	56 (9.5%)
30–39	68 (11.5%)
40–49	114 (19.3%)
50–59	123 (20.8%)
60–69	154 (26.1%)
70–89	75 (12.7%)
**Educational level**	
Elementary school	3 (0.5%)
High school	174 (29.5%)
College	196 (33.2%)
Bachelor's degree	163 (27.6%)
Master's or doctorate degrees	54 (9.2%)
**Household composition**	
Living alone	120 (20.3%)
Living together	470 (79.7%)
**Childcare**
No children at home due to COVID-19	506 (85.8%)
Children at home due to COVID-19	84 (14.2%)
**Health problem**	
No COVID-19-specific health risk	359 (60.8%)
One or more COVID-19-specific health risk	231 (39.2%)
**Mental health problems**
Yes	80 (13.6%)
No	510 (86.4%)
**COVID-19 questions**	
Know someone diagnosed with COVID-19 (Yes)	77 (13.1%)
Essential worker with greater risk of exposure (Yes)	96 (16.3%)
Healthcare worker	36 (6.1%)
**Psychiatric symptoms**	
Depression (PHQ-2 ≥ 3)	69 (11.7%)
Anxiety (GAD-2 ≥ 3)	107 (18.1%)

*PHQ-2, Patient Health Questionnaire-2; GAD-2, Generalized Anxiety Disorder-2*.

### Initial Exploratory Factor Analysis

The normality assumption for each item on the SAVE-6 scale was checked based on skewness and kurtosis within a range of ± 2 (Table 2). The KMO measure of 0.80 and Bartlett's test of sphericity of *p* < 0.001 showed that these data were suitable for factor analysis. A screen analysis showed that the French–Canadian version of the SAVE-6 scale was clustered into single factors based on an eigenvalue plot over 1 (eigenvalue = 2.79) with acceptable model fits (RMS*R* = 0.057, RMSEA = 0.097). Results from the parallel analysis also provided support for the single structure model (reduced eigenvalue = 2.143, 95 percentile of random reduced eigenvalue = 0.215).

**Table 2:** Factor structure of the SAVE-6 scale and factor loadings.

**Items**	**Responses (%)**					**Mean ±SD**	**Skewness**	**Kurtosis**	**CITC**	**CID**	**Factor loading (95% CI)**
	**0**	**1**	**2**	**3**	**4**						
1. Are you afraid the virus outbreak will continue indefinitely?	14.4	19.7	38.6	20.7	6.6	1.85 ± 1.11	−0.06	−0.61	0.33	0.771	0.36 (0.29, 0.42)
2. Are you afraid your health will worsen because of the virus?	25.3	31.0	29.5	10.2	4.1	2.37 ± 1.09	0.46	−0.42	0.51	0.726	0.59 (0.51, 0.67)
3. Are you worried that you might get infected?	13.6	26.6	39.0	16.3	4.6	1.72 ± 1.04	0.10	−0.45	0.65	0.690	0.79 (0.70, 0.88)
4. Are you more sensitive toward minor physical symptoms than usual?	9.2	19.8	28.1	30.2	12.7	2.18 ± 1.16	−0.21	−0.80	0.54	0.718	0.64 (0.57, 0.72)
5. Are you worried that others might avoid you even after the infection risk has been minimized?	13.4	18.6	28.8	27.8	11.4	2.05 ± 1.21	−0.17	−0.88	0.56	0.710	0.68 (0.60, 0.76)
6. Do you worry your family or friends may become infected because of you?	27.8	28.3	27.5	12.4	4.1	1.37 ± 1.13	0.44	−0.64	0.46	0.739	0.50 (0.43, 0.57)

*SAVE-6, Stress and Anxiety to Viral Epidemics-6 items; CITC, corrected item-total correlation; CID, Cronbach's alpha if item deleted; CI, confidence interval*.

*—never; 1—rarely; 2—sometimes; 3—often; 4—always*.

### Confirmatory Factor Analysis

**Figure 1:**
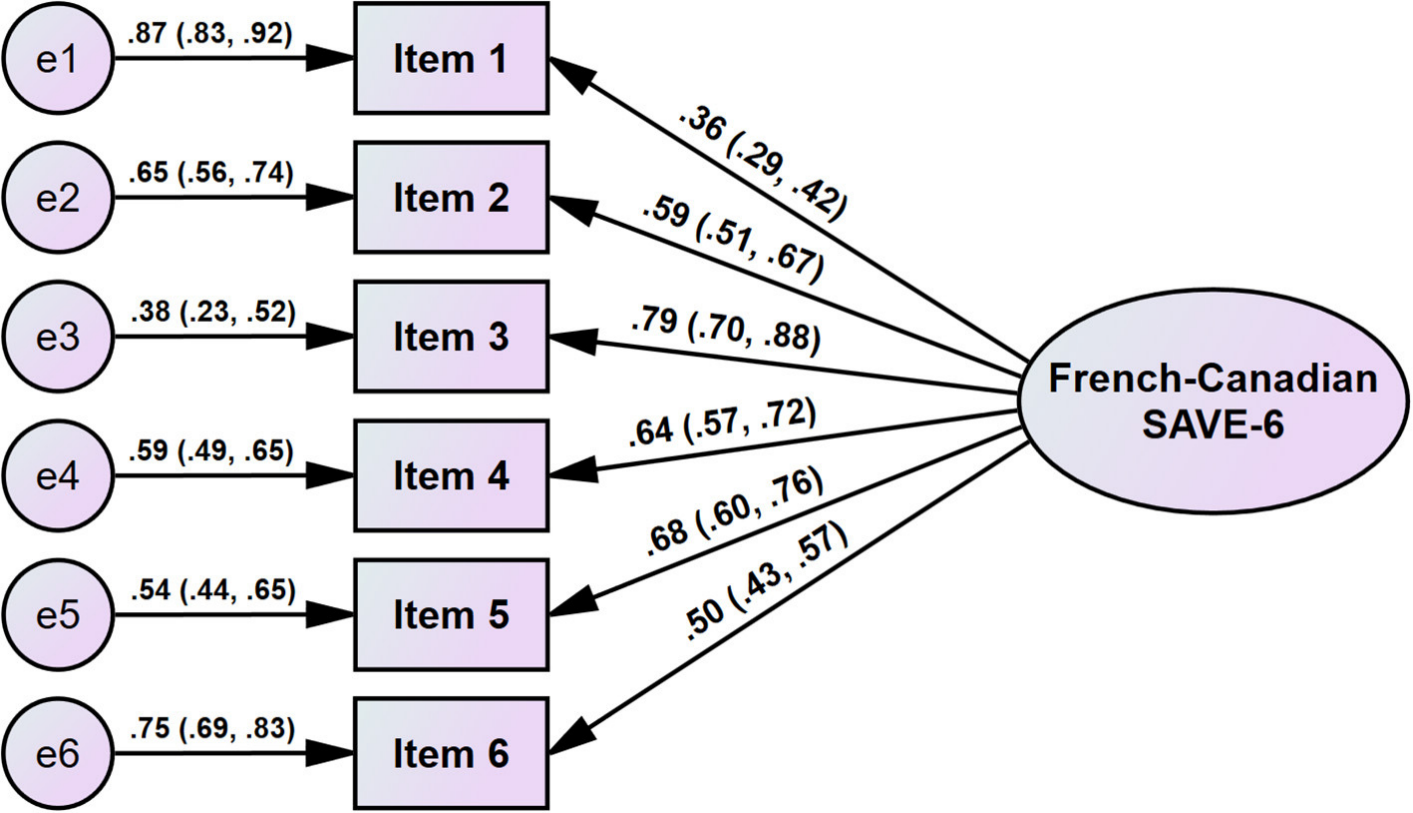
Factor structure of the French–Canadian version of the SAVE-6 scale.

The CFA of the SAVE-6 scale showed a good model fit (CFI = 0.985, TLI = 0.976, RMSEA = 0.051, RSM*R* = 0.048, Table 3) for the single factor structure (Figure 1). Therefore, we could adopt the French–Canadian version of the SAVE-6 scale to assess the anxiety response of the general population to the pandemic. The multi-group CFA revealed that the factor structure of the SAVE-6 scale can measure the anxiety response in the same way among respondents with depression (PHQ-2 ≥ 3, CFI = 0.993, TLI = 0.989, RMSEA = 0.034, RSM*R* = 0.049) and anxiety (GAD-2 ≥ 3, CFI = 0.988, TLI = 0.980, RMSEA = 0.044, RSM*R* = 0.051) and across both genders (male vs. female, CFI = 0.985, TLI = 0.976, RMSEA = 0.051, RSM*R* = 0.056), across respondents who knew or did not know someone diagnosed with COVID-19 (CFI = 0.990, TLI = 0.984, RMSEA = 0.041, RSM*R* = 0.050), and across respondents with and without occupational risk of SARS-CoV-2 exposure (CFI = 0.993, TLI = 0.988, RMSEA = 0.036, RSM*R* = 0.050). Multi-group CFA with metric or scale invariant model also showed similar results.

**Table 3:** Scale level psychometric properties of the French–Canadian version of the SAVE-6 scale.

**Psychometric properties**	**Scores**	**Suggested cutoff**
Floor effect	1.7%	15%
Ceiling effect	0.5%	15%
Mean inter-item correlation	0.35	Between 0.15 and 0.50
Cronbach's alpha	0.76	≥767
McDonald's Omega	0.77	≥777
Split-half reliability (odd-even)	0.81	≥817
Standard error of measurement	2.23	Smaller than SD (4.55)/2
Ferguson delta	0.97	≥979
Loevinger's H coefficients	0.38	–
*Rho* coefficient	0.76	≥767
IRT reliability	0.83	≥837
**Model fits of confirmatory factor analysis**		
*χ^2^* (d*f, p*-value), *χ^2^*/d*f*	22.680 (9, 0.007), 2.52	Nonsignificant, <5
CFI	0.985	>0.95
TLI	0.976	>0.95
RMSEA [90% CI value] (*p*-value)	0.051 [0.025, 0.077] (0.437)	<0.08
SRMR	0.048	<0.08

*SAVE-6, Stress and Anxiety to Viral Epidemics-6 items; SD, standard deviation; IRT, item response theory; df, degree of freedom; CFI, comparative fit index; TLI, Tucker Lewis index; RMSEA, root-mean-square-error of approximation; SRMR, standardized root-mean-square residual; CI, confidence interval*.

### Reliability and Evidence Based on Relations to Other Variables

The French–Canadian version of the SAVE-6 scale showed good internal consistency (Cronbach's alpha = 0.76, McDonald's Omega = 0.77). The Cronbach's alpha if item deleted was 0.69 ~ 0.77. The total score of the SAVE-6 scale was significantly correlated with the severity of depression [PHQ-2 score, *r* = 0.308 (95% CI, 0.233, 0.379), *p* < 0.001] and anxiety [GAD-2 score, *r* = 0.434 (95% CI, 0.366, 0.497), *p* < 0.001]. The SAVE-6 score was observed to be significantly higher among participants with depression [PHQ-2 ≥ 3, *t*(588) = 5.068, *p* < 0.001] and anxiety [GAD-2 ≥ 3, *t*(588) = 8.396, *p* < 0.001]. In addition, the SAVE-6 scale score was higher among females [vs. male, *t*(588) = 2.448, *p* = 0.015], with children at home [*t*(588) = 2.429, *p* = 0.015], or knowing someone diagnosed with COVID-19 [*t*(588) = 3.170, *p* = 0.002], but it was not greater among those living with other people [*t*(588) = 1.342, *p* = 0.180] or at greater occupational risk of exposure to SARS-COV-2 [*t*(588) = 1.449, *p* = 0.148].

### Psychometric Properties of the French–Canadian Version of the Stress and Anxiety to Viral Epidemics-6 Items Scale

Psychometric properties of the French–Canadian version of the SAVE-6 scale were also assessed by utilizing the GRM (an IRT model for polytomous items). Loevinger's *H* coefficient (0.38; Table 3) suggests that this scale is unidimensional, but only weakly. Non-significant *p*-values of G2 (Supplementary Table 1) suggest the absence of local dependency between the items of the SAVE-6 scale. Supplementary Table 2 shows that there is a significant violation of monotonicity in item 1 and two significant violations in item 5. However, crit values (56 for item 1 and 53 for item 5) suggest unclear evidence of violation (37). Therefore, we can come into a conclusion that all the assumptions for the IRT model (unidimensional and local dependence) are met. RMSEA values for *S*_χ^2^ are ranged between 0.000 and 0.048 (Supplementary Table 2), that are below the recommended cut off for the RMSEA (<0.08). This result suggests that all the items belong to the French–Canadian version of the SAVE-6. Supplementary Table 1 demonstrates the slope/discrimination parameters (α) and threshold/difficulty parameters (*b*). Slope parameters ranged between 0.799 and 3.032 (mean = 1.690). The slopes for items 1 and 5 were moderate, the slope of item 2 was high, and the slopes of the rest of the items were very high. These values indicate the efficiency in discrimination among individuals in this latent trait. Results regarding threshold coefficients (*b*) suggest that a higher latent trait or theta is required to endorse items 2 and 6 compared to other items. In items 2 and 6, only *b*1 coefficients were negative and the rest were positive. This suggests that an above-average level of latent trait or theta is required to endorse Likert-type response options—from “sometimes” to “often.” Item information curves in Supplementary Figure 1 graphically depict this information. The scale information curve in Supplementary Figure 2 provides an understanding of the information provided by the French–Canadian SAVE-6 scale. From this curve, this scale provides more information about people between the −1.25 and 1.90 θ level. There are several peaks in the curve; these might be due to the polytomous nature of the data.

## Discussion

In this study, we observed a good model fit for the single structure of the French–Canadian version of the SAVE-6 scale and a good convergent validity with a previously validated rating scale (PHQ-4). The SAVE-6 scale was observed to be a reliable and valid rating scale for measuring the general populations' anxiety responses to the viral epidemic.

The SAVE-6 scale was drawn from the original SAVE-9 scale, which was developed for assessing the work-related stress and anxiety responses of frontline healthcare workers to the viral COVID-19 pandemic (26). The SAVE-9 scale was clustered into two factors: factor I (items 1, 2, 3, 4, 5, and 8) and factor II (items 6, 7, and 9). Since this clustering is parallel to the other validation studies of scales translated into the Italian (38), Japanese (39), and Turkish (40) languages, we chose to apply factor I of the SAVE-9 scale, namely SAVE-6, as a tool for measuring the anxiety responses of the general population to this pandemic (18). In this study, the construct validity is comparable to those in the previous studies conducted among Korean (18) and Lebanese (19) general population samples. The convergent validity of the SAVE-6 scale is in accordance with the PHQ-9 or GAD-7, and the SAVE-6 scale is a valid tool for measuring the anxiety symptoms in this pandemic era. Unfortunately, we did not apply the GAD-7 scale in this study, and we could not provide the optimal cut-off score of the SAVE-6 in accordance with the 5 points of the GAD-7 (mild degree of general anxiety). In previous studies, 12–16 point of cut-off scores for the SAVE-6 scale were reported among various samples (18, 19, 21, 22).

In this study, we found that the French–Canadian version of the SAVE-6 scale could reliably measure the anxiety responses of the general population. Interestingly, the SAVE-6 scale score was higher among females or participants who knew someone diagnosed with COVID-19. During the COVID-19 pandemic, the level of stress or anxiety related to the viral epidemic was higher among women compared to men in the general population (41, 42), and this was true even among healthcare workers (6, 43). While developing a new rating scale, Silva et al. (17) and Chung et al. (18) observed a higher level of anxiety among female participants. This ought to be considered when developing an anxiety scale targeting the viral epidemic. The level of anxiety was higher among participants who knew someone diagnosed with COVID-19. In our previous study involving the American sample (20), viral anxiety was significantly higher among participants who knew someone who died of COVID-19. Although there is a discrepancy between death due to COVID-19 and being diagnosed with COVID-19, it indicates that when an acquaintance gets infected, it might influence one's own anxiety.

However, viral anxiety was not significantly higher among essential workers who are at a greater risk for occupational exposure to SARS-CoV-2. We can speculate that participants who were not working in conditions that put them at a higher risk of COVID-19 also suffer from viral anxiety since the survey was conducted from September 28 to October 18, 2020, when most people were at higher risk of COVID-19 infection. Further, the SAVE-6 scale score did not differ among participants who were living with other people and those who were not. However, the SAVE-6 score was significantly higher among participants with children at home. These results are parallel to a study conducted in the United Kingdom, which showed that those with children at home and adults who were not alone were associated with anxiety or depression (44).

The limitations of this study are, first, that it was based on an online survey, rather than a process of interviewing participants face-to-face, and this could possibly lead to biased data. This was necessary to prevent the spread of the virus during this pandemic. We therefore gathered responses from various areas of Quebec without face-to-face contact with the participants. Second, the survey participants were part of a web panel, which could lead to a bias of self-selection. Moreover, the participants in this validation study were part of another longitudinal study, which was conducted to explore the factors for adherence to physical distancing during COVID-19. This also might lead to selection bias. Third, we could not gather information on the participants' experiences of quarantine due to being infected with COVID-19. In conclusion, the French–Canadian version of the SAVE-6 scale is a valid and reliable rating scale, which can measure the general population's anxiety responses to the viral epidemic.

## Data Availability Statement

The raw data supporting the conclusions of this article will be made available by the authors, without undue reservation.

## Ethics Statement

The studies involving human participants were reviewed and approved by Concordia University Institutional Ethics Review Board. Written informed consent for participation was not required for this study in accordance with the national legislation and the institutional requirements.

## Author Contributions

SC and SS: conceptualization and resources. J-PG: data curation. SC and OA: formal analysis. SL, SS, and CHKP: investigations. SC, SS, OA, and J-PG: methodology. CHKP and SL: project administration. OA and CHKP: visualization. OA, CHKP, and SC: writing—original draft. All authors writing—review and editing.

## Funding

This work was supported under the Framework of International Cooperation Program managed by the National Research Foundation of Korea [FY2020K2A9A1A01094956]. The funding source was not involved in the study design; in the collection, analysis, or interpretation of data; in the writing of the report; or in the decision to submit the article for publication.

## Conflict of Interest

The authors declare that the research was conducted in the absence of any commercial or financial relationships that could be construed as a potential conflict of interest.

## Publisher's Note

All claims expressed in this article are solely those of the authors and do not necessarily represent those of their affiliated organizations, or those of the publisher, the editors and the reviewers. Any product that may be evaluated in this article, or claim that may be made by its manufacturer, is not guaranteed or endorsed by the publisher.

## Supplementary Material

Supplementary Figure 1 Scale information curve of the French–Canadian version of the SAVE-6 scale.

Supplementary Figure 2 Item response category characteristic curve for each item of the French–Canadian version SAVE-6 scale.

Supplementary Table 1 Local dependence G2 p-values, and slope and threshold parameters of the graded response model.

Supplementary Table 2 Monotonicity and item fit results of the French-Canadian version of the SAVE-6.

## Abbreviations

CFA: Confirmatory Factor Analysis
SAVE-6: Stress and Anxiety to Viral Epidemics-6 items
IRT: Item Response Theory
PHQ-4: Patient Health Questionnaire-4 items
PHQ-9: Patients Health Questionnaire-9
GAD-7: Generalized Anxiety Disorder-7
GAD-2: generalized anxiety disorders screener
MRFA: Minimum Rank Factor Analysis
KMO: Kaiser-Meyer-Olkin
SRMR: standardized root-mean-square residual
RMSEA: root-mean-square-error of approximation
CFI: comparative fit index
TLI: Tucker Lewis index
GRM: graded response model.
