# Supplementary figures and images for: The Psychometric Properties of the French–Canadian Stress and Anxiety to Viral Epidemics-6 Scale for Measuring the Viral Anxiety of the General Population During the COVID-19 Pandemic
Source: Front Psychiatry. 2022 Mar 31;13:807312. doi: 10.3389/fpsyt.2022.807312 (PMC9008890; doi:10.3389/fpsyt.2022.807312)

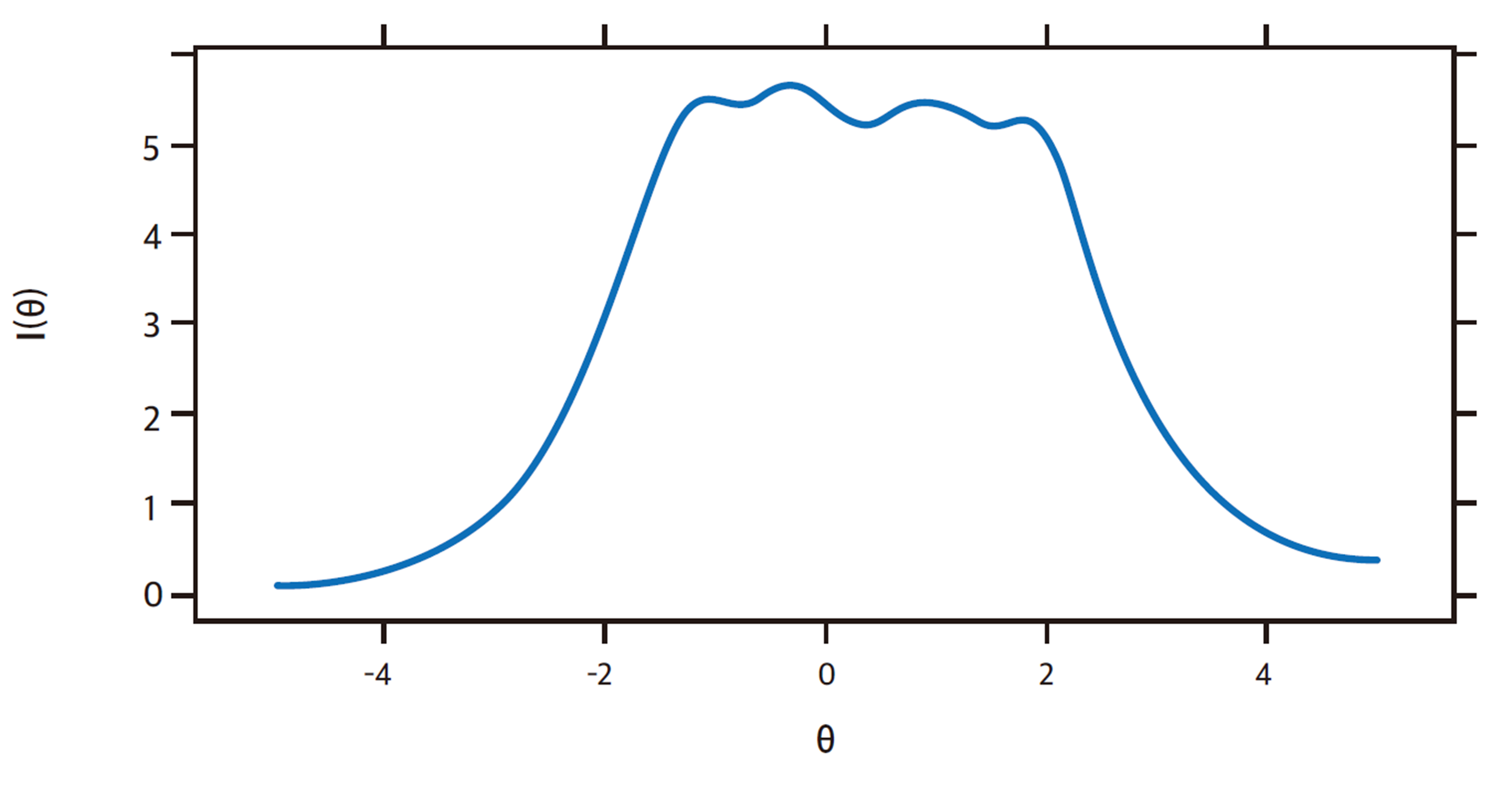

Supplement: Supplementary Figure 1 — Scale information curve of the French–Canadian version of the SAVE-6 scale. [file Image_1.TIF]

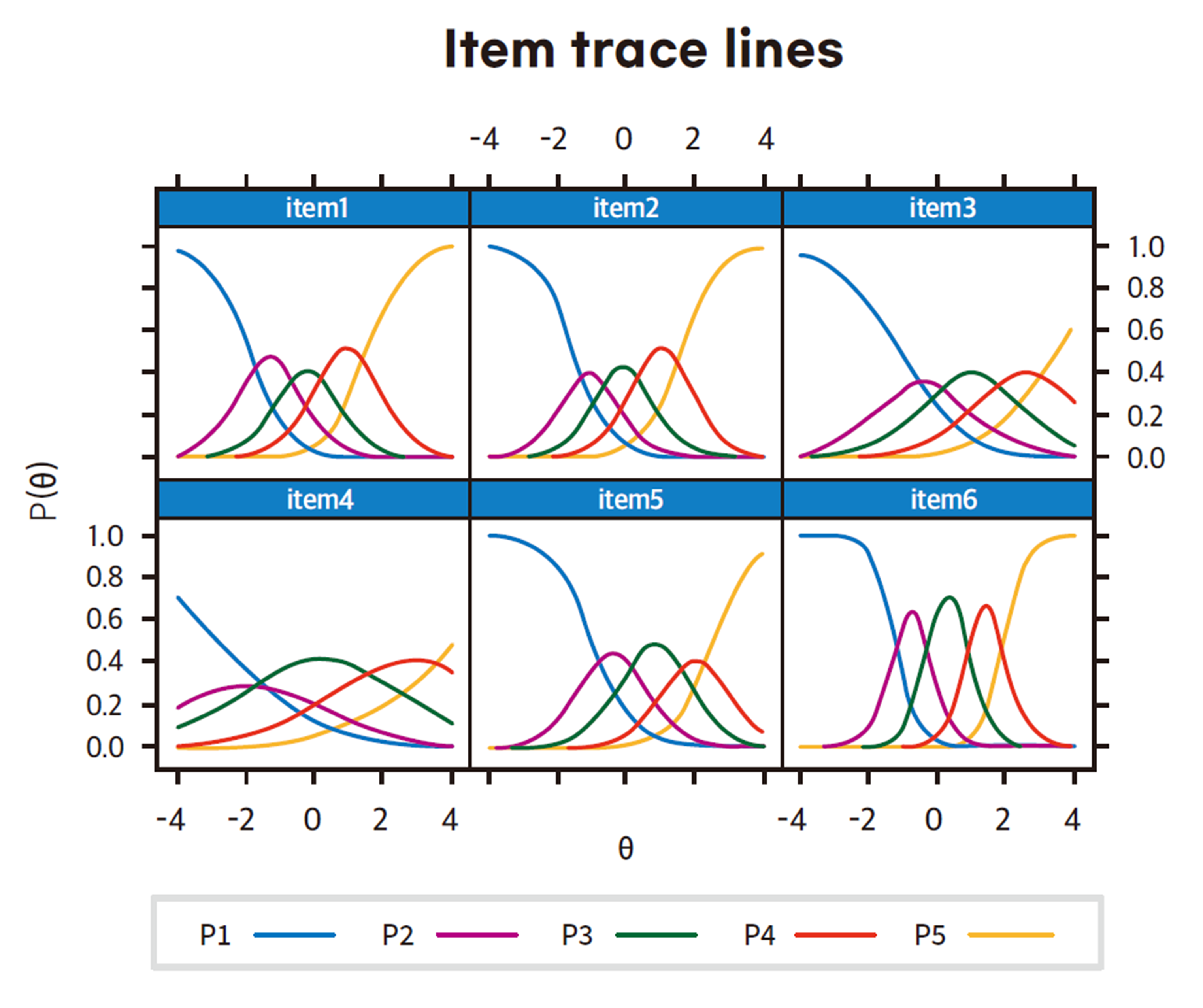

Supplement: Supplementary Figure 2 — Item response category characteristic curve for each item of the French–Canadian version SAVE-6 scale. [file Image_2.TIF]
