# Supplementary material for: The Psychometric Properties of the French–Canadian Stress and Anxiety to Viral Epidemics-6 Scale for Measuring the Viral Anxiety of the General Population During the COVID-19 Pandemic
Source: Front Psychiatry. 2022 Mar 31;13:807312. doi: 10.3389/fpsyt.2022.807312 (PMC9008890; doi:10.3389/fpsyt.2022.807312)
Supplement: Supplementary Table 1 — Local dependence G2 p-values, and slope and threshold parameters of the graded response model. [file Data_Sheet_1.docx]

**Supplementary Table 1**. Local dependence G2 *p* values, and slope and threshold parameters of the graded response model

| **Items** | **Local dependence G^2^ *p* values** | | | | | **Slope parameter (α)** | **Threshold parameter (b)** | | | |
| --- | --- | --- | --- | --- | --- | --- | --- | --- | --- | --- |
|  | **Item 1** | **Item 2** | **Item 3** | **Item 4** | **Item 5** |  | **b_1_** | **b_2_** | **b_3_** | **b_4_** |
| Item 1 |  |  |  |  |  | .799 | -2.758 | -1.026 | 1.539 | 4.075 |
| Item 2 | .199 |  |  |  |  | 1.627 | -.947 | .212 | 1.517 | 2.582 |
| Item 3 | .199 | .199 |  |  |  | 3.032 | -1.247 | -.266 | .896 | 1.944 |
| Item 4 | .199 | .199 | .199 |  |  | 1.738 | -1.883 | -.709 | .285 | 1.604 |
| Item 5 | .199 | .199 | .199 | .199 |  | 1.871 | -1.502 | -.587 | .396 | 1.634 |
| Item 6 | .199 | .199 | .199 | .199 | .238 | 1.070 | -1.100 | .245 | 1.777 | 3.384 |

*P* values adjusted for false discovery rate

**Supplementary Table 2.** Monotonicity and item fit results of the French-Canadian version of the SAVE-6

| **Items** | **Monotonicity** | | | | **Item fits** | | |
| --- | --- | --- | --- | --- | --- | --- | --- |
|  | **ac** | **Vi** | **zsig** | **crit** | **S_χ^2^** | **df_ S_χ^2^** | **RMSEA_ S_χ^2^** |
| **Item1** | 81 | 7 | 1 | 56 | 47.132 | 51 | .000 |
| **Item2** | 72 | 2 | 0 | 21 | 63.991 | 41 | .031 |
| **Item3** | 51 | 1 | 0 | 8 | 28.867 | 28 | .007 |
| **Item4** | 112 | 3 | 0 | 14 | 38.371 | 38 | .004 |
| **Item5** | 84 | 4 | 2 | 53 | 91.479 | 39 | .048 |
| **Item6** | 52 | 3 | 0 | 22 | 75.769 | 45 | .034 |
| ac = active comparison, vi = violation, zsig = significant violation | | | | | | | |
